# Patient experience in retinitis pigmentosa and Choroideremia– a concept elicitation study in 17 patients based on qualitative interviews

**DOI:** 10.1186/s13023-025-03713-4

**Published:** 2025-08-11

**Authors:** Elke Rometsch, Per-Olof Thuresson, Noémie Hurst, Eckhart Eule, Isabel Bachmeier, Benoit Blanchard, José-Alain Sahel, Isabelle Audo

**Affiliations:** 1 F. Hoffmann-La Roche Ltd, Basel, Switzerland; 2 DHU Sight Restore, Centre Hospitalier National d’ophtalmologie des Quinze-Vingts, Centre de Référence Maladies Rares REFERET, INSERM-DHOS CIC 1423, Paris, France; 3 Institut de la Vision, Sorbonne Universités, INSERM, CNRS, Paris, France; 4 Department of Ophthalmology, School of Medicine, University of Pittsburgh, Pittsburgh, PA USA

**Keywords:** Patient-reported outcomes, Qualitative research, Interview, Retinitis pigmentosa, Choroideremia, Conceptual model, Disease experience, Symptom burden, Activities of daily living, Quality of life

## Abstract

**Background:**

Retinitis pigmentosa (RP) and Choroideremia (CHM) are rare inherited retinal diseases (IRDs) that can lead to severe visual impairment or blindness. Despite different underlying genetic pathways, they have many progression patterns in common, first affecting the more peripheral retina and later advancing toward the central retina. Early symptoms of both diseases include night blindness, difficulty adjusting to changing levels of light, and difficulty seeing in poor contrast. This is followed by progressive loss of the peripheral visual field in daylight, and eventually blindness. Currently, hardly any studies based on interviews that describe symptom experience and impact exist. In this concept elicitation study, we conducted qualitative interviews to identify the symptoms experienced by RP (*n* = 12) and CHM (*n* = 5) patients, and to understand the impact of symptoms on patients’ lives.

**Results:**

Among the 14 symptoms reported, poor night vision/night blindness, difficulty seeing in bright light, and difficulty seeing in low/dim light were experienced by all participants. Over 50% of participants in either condition reported difficulty adapting from bright to dark and vice versa, poor peripheral vision, poor contrast sensitivity, poor distance vision, and poor visual acuity. Symptoms had a significant impact on activities of daily living. Most commonly impacted were the ability to navigate and the use of digital screens (*n* = 17/17, 100%) as well as physical functioning and work/school-related activities (*n* = 16/17, 94.1%). These impacts were often exacerbated by environmental factors. For instance, all participants reported this for navigation. Additionally, most participants (*n* = 13/17, 76.5%) reported impacts on emotional well-being. Concept saturation was shown for the RP sample (*n* = 12) and the combined sample (*n* = 17), i.e., all concepts reported were spontaneously mentioned in the first 3 sets of interviews. This suggests that further interviews would be unlikely to yield any new symptom concepts.

**Conclusion:**

The patient interviews provided insight into the patient experience of RP and CHM and formed the basis for developing a combined conceptual model of RP and CHM disease experience, suitable to serve as basis for future patient reported and performance outcome measures. Testing in larger samples, especially in CHM, is recommended to further evaluate content validity of these preliminary findings.

**Supplementary Information:**

The online version contains supplementary material available at 10.1186/s13023-025-03713-4.

## Background

Retinitis Pigmentosa (RP) and Choroideremia (CHM) are inherited retinal diseases (IRDs) that can lead to severe visual impairment or blindness. While RP is the most common IRD, with a total prevalence of approximately 1 in 4,000 [1], it is a heterogeneous group of rod-cone dystrophies with different genes affected, resulting in different prevalences, inheritance patterns, progression rates and prognoses. CHM is an even rarer disease with a prevalence of 1 in 50,000 to 1 in 100,000 individuals [2]. Despite the different underlying genetic pathways between these disorders and the slightly different evolution, they have many progression patterns in common. Both conditions first affect the more peripheral retina and later advance toward the central retina. One of the first symptoms of both RP and CHM is night blindness, followed by progressive loss of the peripheral visual field in daylight, eventually leading to blindness.

In addition, people with RP and CHM often exhibit difficulty adjusting to changing levels of light, difficulty seeing in poor contrast, and motion perception anomalies [3, 4].

Assessment of disease severity and progression relies on clinical funduscopic examination, multimodal retinal imaging, and electrophysiological tests, along with other functional assessments. The latter have historically been focused on traditional outcome measures (e.g., visual acuity, visual field) [5]. While such functional assessments are important, they do not convey other important aspects of vision that are impaired in RP and CHM patients, such as mesopic and scotopic vision, light sensitivity, adaptation to different lighting conditions, contrast sensitivity, and navigation. Furthermore, these measures alone are of limited value in assessing the impact of vision-specific symptoms on activities of daily living (ADL), functional vision, and health-related quality of life (HRQOL).

Therefore, there is a high unmet need for clinical outcome assessments (COA) measuring symptoms closer to patients’ experience and needs [6].

COA development starts with qualitative research (e.g., one-on-one interviews and focus groups) to understand which concepts matter the most to patients living with a disease or condition [7]. Concept elicitation is the process of identifying the symptoms experienced and the functions affected as the result of a given disease and how this has an impact on patients’ lives. This is typically done through semistructured qualitative interviews with patients. Only two studies on qualitative interviews with patients suffering from RP or CHM had been published at the start of this project [3, 8]. These publications still left questions open concerning the relative importance of different symptoms for patients, the impact of these symptoms along with environmental conditions on patients’ ADL, and the correlation of disease severity with the level of impact on ADL, which are key elements in the development of new COAs, such as patient-relevant patient-reported outcomes (PRO) and performance outcomes (PerfO).

Therefore, this study aimed to (1) assess the link between vision impairment, disease severity, and impact on ADL in RP and CHM; (2) assess the impact of environmental variables (e.g., light condition) on the ability to perform ADL in RP and CHM of different disease severities; (3) identify the most relevant impairments of ADL in patients with RP and CHM of different disease severities; and (4) identify what visual functions are impaired as a result of RP and CHM. Ultimately, this information should serve to inform the development of new patient-relevant PROs and PerfOs for RP and CHM.

## Methods

**Fig. 1:**
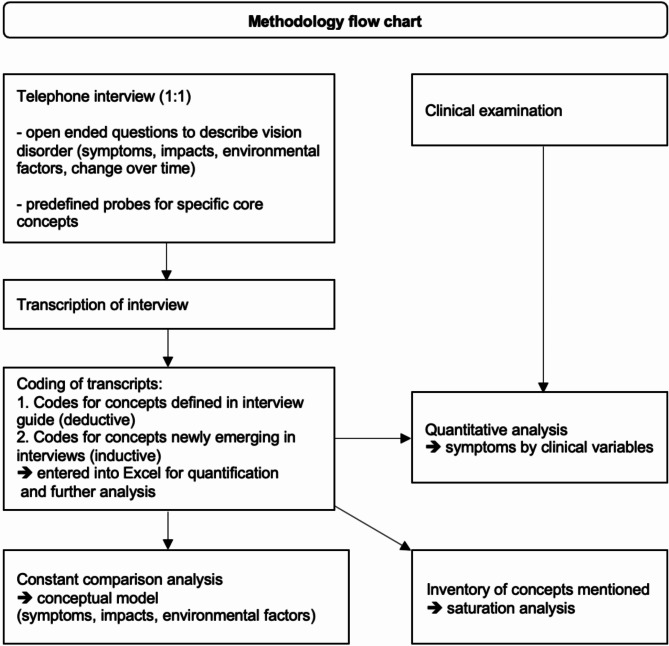
Methodology flow-chart

An overview of the study methodology is depicted in Fig. 1.

This was a single site, non-interventional, cross sectional qualitative interview study in patients suffering from RP (*n* = 12) and CHM (*n* = 5). A total of 12 participants were identified as the number needed to reach conceptual saturation in a relatively homogeneous population [9, 10] and were thus deemed adequate to ensure the identification of concepts experienced by patients with RP, whereas the identification of concepts for CHM was secondary due to feasibility.

Participants were reimbursed with 30 Euros for their time and only interviewed after they provided verbal and written informed consent.

Staff from the Rare Disease Centre RefeRet of Centre Hospitalier National d’Ophtalmologie (CHNO) des Quinze-Vingts (Paris, France) reviewed the clinical records of their existing RP and CHM patients to identify potentially eligible interview participants. Of the 150 records pre-screened, 20 patients were identified as potentially eligible. These patients were asked whether they were interested to participate in the study. Two refused, so 18 patients signed informed consent and were screened. One patient was a screening failure, leaving 17 participants. A specialized agency (Adelphi Values, Bollington, UK) and their partner fieldwork agency (Zeste Research, Looses, France) then coordinated and conducted the interviews. To ensure adequate representation of participants across disease severities, quotas were used upon enrollment.

### Conduct of interviews and coding

All interviews were conducted by telephone between July 2021 and January 2022. A trained and experienced qualitative interviewer interviewed the participants in 1:1 interviews for approximately 60 min in French language following an interview guide. Patients were asked to describe their experience of vision disorder, including the symptoms and impact of the symptoms, the impact of environmental conditions (e.g., lighting) on ADL, the reasons for seeking medical help for their ocular symptoms, and any changes in their symptoms over time. To allow spontaneous mention of concepts, this was done by asking open-ended questions.

The interviewers were instructed to probe for specific core concepts (Table 1) that had not been spontaneously reported by the participants during the initial open-ended questioning. The core symptoms and vision problems were identified in a literature review conducted for this purpose. The discussion of core symptoms was followed by additional questions about the specific ways individual participants were impacted.

**Table 1:** Core symptoms/vision problems defined in the interview guide

Color blindness
Difficulty in bright light
Difficulty in low/dim light
Difficulty adapting to light changes: from dark to light
Difficulty adapting to light changes: from bright to dark
Poor distance vision
Loss of central vision
Poor contrast sensitivity
Poor night vision/night blindness
Poor peripheral vision (e.g., difficulty noticing objects at the side, narrowing of vision, “tunnel vision”)
Visual acuity

For some characteristics of symptoms, some impacts, as well as environmental conditions, the interview guide suggested nonmandatory follow-up topics (Tables 9 and 10– supplement). Since the interview guide was not to be used as a fixed script but merely to guide the discussion, the order and depth in which the concepts were covered varied in the individual interviews.

The interviews were then transcribed verbatim and translated to English by TransPerfect (New York, United States). The interview transcripts were entered into Atlas.ti (version 8, ATLAS.ti Scientific Software Development GmbH, Berlin Germany) for coding and analysis. Our analysis comprised both deductive and inductive coding. First, we created deductive codes based on the interview guide. Second, we assigned inductive codes to the themes that appeared important but had not yet been covered, i.e., themes that newly emerged during the interviews [11].

The coded segments were extracted to Microsoft Excel (MSOffice 16; Version 2180, Microsoft Corporation, Redmond, Washington, United States) to enable generating numerical scores to determine their prevalence, i.e., the number of participants mentioning the respective topic [12, 13]. Examples of how codes were derived from the interviews are depicted in Table 11 in the supplement.

### Population

To be included in the study, patients had to be ≥ 13 years old, diagnosed with genetically confirmed nonsyndromic bilateral RP or CHM, with no other overlapping retinal disease and clear ocular media. Patients had to be willing and able to understand and respond to the interview in French and had to have at least a best corrected visual acuity (BCVA) ≥ 55 ETDRS letter equivalent in one or both eyes and a binocular Goldmann visual field of ≥ 10°. This threshold was chosen because we did not want to focus on patients facing challenges of ultra-low vision. We also excluded patients for whom the time of disease symptom onset was unknown and patients suffering from any other condition that could affect their visual function (e.g., glaucoma, high myopia, recent eye surgery) or their motor skills (e.g., inability to walk).

**Fig. 2:**
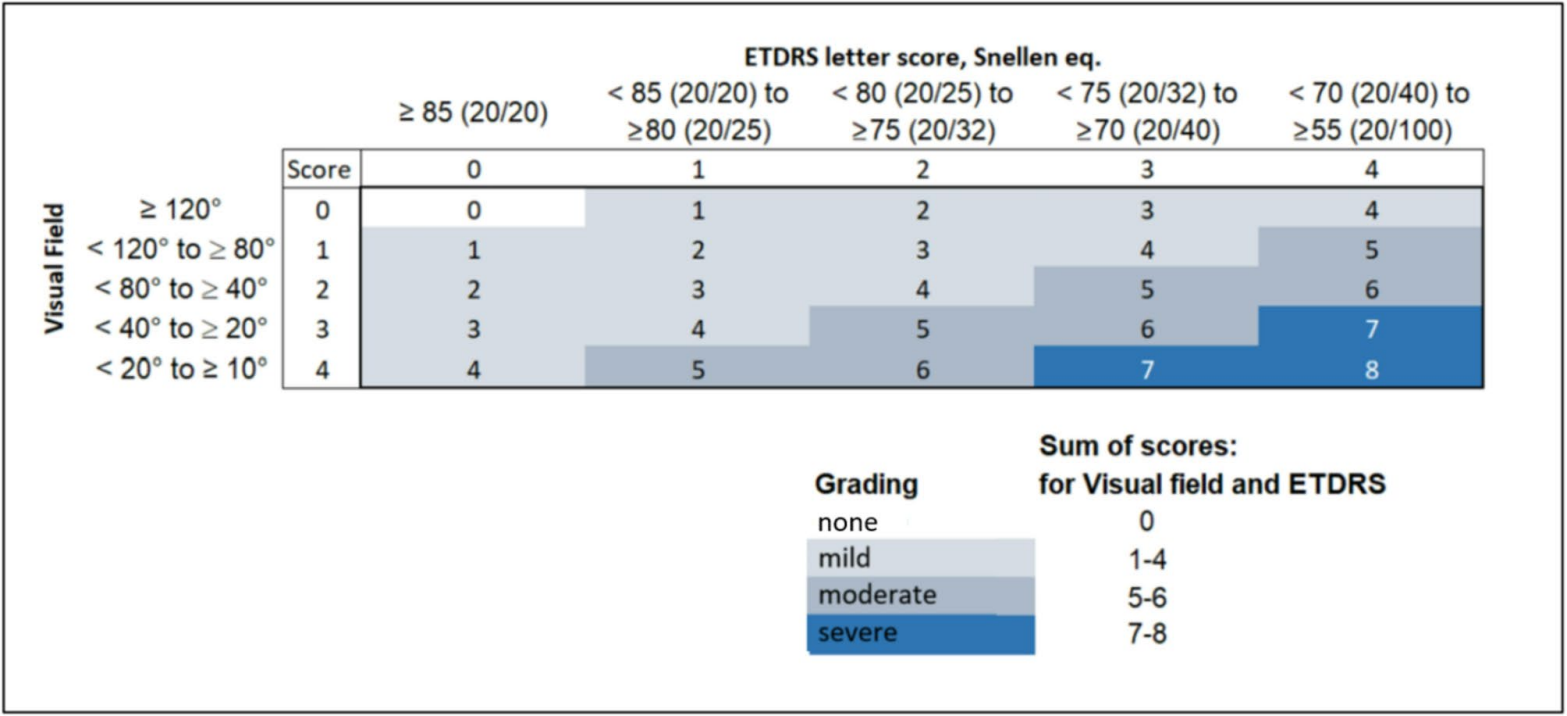
Simplified grading of disease severity for RP and CHM

To enable recruiting a balanced sample of patients across different diseases and disease severities, we assessed disease characteristics before enrolling the patients. This assessment was a simplification of the Iftikhar grading system, which is based on both clinical and imaging data and was originally developed for RP [14]. This simplification (Fig. 2), based on visual acuity and visual field alone, was needed because at the beginning of the study during the COVID-19 pandemic, optical coherence tomography (OCT) was not available for all participants. Once OCTs became available, we determined the width of the ellipsoid zone and then used the original Iftikhar classification, which calculates a cumulative score that is then categorized into grades, for the analysis.

For CHM, no comparable grading system exists. Based on the similarity of symptoms experienced by RP and CHM patients [15–20], we applied the same classification to characterize both RP and CHM patients.

### Analysis

Sociodemographic information and disease severity as well es the prevalence of the concepts mentioned by the participants were summarized with descriptive statistics.

Conceptual saturation is generally determined based on spontaneous reports of concepts only [9, 10]. It is deemed as achieved when researchers can show that they have covered the topic of interest in depth by having a sufficient sample to explore the concepts fully. In other words, when further analysis or new data collection adds no new concept-relevant information, it is commonly assumed that conceptual saturation has been achieved. We determined conceptual saturation for the total sample and for RP alone by checking whether new themes had emerged after each set of interviews.

The interview sets were defined by grouping all interviews into sets of 3 (for RP) and 4 or 5 (for the complete sample) in the order in which they had been conducted [9, 10].

We used constant comparison analysis [21] to develop the conceptual model, which includes symptoms, impacts on ADLs, and environmental exacerbating factors.

Concept frequency, i.e., the number of patients who mentioned a specific concept in their interview, was calculated for all symptoms and impacts, including environmental exacerbating factors, for the total sample and separately for RP and CHM. The clinical parameters BCVA, ellipsoid zone, Iftikhar cumulative score, and Goldmann visual field (GVF) as well as the total sum of symptoms were analyzed according to the presence or absence of individual symptoms.

## Results

### Population

The total study population included 12 participants with RP and 5 participants with CHM. The total study population had a median (Q1; Q3) age of 36 (28; 54) years and comprised 9 women (52.9%) and 8 men (47.1%) (Table 1). Most participants had experienced their first symptoms at a median (Q1; Q3) time of 6 (4; 13) years prior to their interview. The disease severity (simplified classification) was assessed as mild in seven patients (*n* = 7/17, 41.2%), moderate in four (*n* = 4/17, 23.5%), and severe in six (*n* = 6/17, 35.3%). The full sociodemographic information and clinical characteristics are shown in Tables 2 and 3.

**Table 2:** Sociodemographic information of the study participants

Sociodemographic information		RP (*n* = 12)	CHM (*n* = 5)	Total (*n* = 17)
Age (years)	average (SD)	36.5 (15.4)	49.6 (20.1)	40.3 (17.4)
	median (Q1;Q3)	32 (25.3;48.8)	43 (36;62)	36 (28;54)
Sex (n, %)	Female	9 (75.0%)	-	9 (52.9%)
	Male	3 (25.0%)	5 (100%)	8 (47.1%)
Level of education (n, %)	Some high school	2 (16.7%)	1 (20.0%)	3 (17.6%)
	High school graduate	4 (33.3%)	1 (20.0%)	5 (29.4%)
	College graduate	6 (50.0%)	2 (40.0%)	8 (47.1%)
	Post graduate degree	-	1 (20.0%)	1 (5.90%)
Work status (n, %)	Retired	-	2 (40.0%)	2 (11.8%)
	Working full time	4 (33.3%)	2 (40.0%)	6 (35.2%)
	Working part time	2 (16.7%)	-	2 (11.8%)
	Student	2 (16.7%)	-	2 (11.8%)
	Unemployed	4 (33.3%)	1 (20.0%)	5 (29.4%)
Driving license (n, %)	Yes	6 (50.0%)	5 (100%)	11 (64.7%)
	No	6 (50.0%)	-	6 (32.3%)
Living area (n, %)	Urban	11 (91.7%)	4 (80.0%)	15 (88.2%)
	Rural	1 (8.30%)	1 (20.0%)	2 (11.8%)

**Table 3:** Clinical characteristics of the study participants

Clinical characteristics		RP (*n* = 12)	CHM (*n* = 5)	Total (*n* = 17)
Years since first symptoms noticed	average (SD)	7 (4.60)	8.3 (4.64)	7.9 (4.51)
	median (Q1;Q3)	7.50 (4.00;13.0)	5.00 (5.00;9.00)	6.00 (4.00;13.0)
Gene mutated** (n, %)	PRPF31 (autosomal dominant)	7 (58.3%)	-	7 (41.2%)
	RP1 (autosomal dominant)	2 (16.7%)	-	2 (11.8%)
	CRB1 (autosomal recessive)	2 (16.7%)	-	2 (11.8%)
	RPE65 (autosomal recessive)	1 (8.30%)	-	1 (8.30%)
	CHM (X-linked)	-	5 (100%)	5 (29.4%)
Disease severity: simplified assessment (n, %)	mild	4 (33.3%)	3 (60.0%)	7 (41.2%)
	moderate	4 (33.3%)	-	4 (23.5%)
	severe	4 (33.3%)	2 (20.0%)	6 (35.3%)
BCVA*[ETDRS letters]	average (SD)	69.8 (8.76)	78.2 (10.6)	72.3 (9.80)
	median (Q1;Q3)	72 (61.2;78.3)	82 (69;87)	73 (65;79)
Goldman Visual Field [°] (binocular, III4)	average (SD)	85 (60.1)	53.2 (39.1)	75.6 (55.6)
	median (Q1;Q3)	70 (29.8;147.8)	44 (23;93)	55 (29;104)
Ellipsoid Zone width [°]	average (SD)	4.2 (3.46)	4.82 (2.66)	4.38 (3.18)
	median (Q1;Q3)	3.90 (1.12;7.38)	4.90 (3.00;5.10)	4.10 (2.10;7.10)
Disease severity: Iftikhar cumulative score	average (SD)	8.92 (2.49)	8.6 (4.16)	8.82 (2.93)
	median (Q1;Q3)	9 (7.50;10.25)	7.00 (5.00;12.0)	8.00 (6.00;11.0)
Disease severity grouping: Iftikhar cumulative score (n, %)	≤ 8	6 (50%)	3 (60%)	7 (41.2%)
	> 8	6 (50%)	2 (40%)	8 (47.1%)
Disease severity: Iftikhar grade	≤ 1	0	0	0
	2	2 (16.7%)	2 (40.0%)	4 (23.5%)
	3	4 (33.3%)	1 (20.0%)	5 (29.4%)
	4	6 (50.0%)	1 (20.0%)	7 (41.2%)
	5	0	1 (20.0%)	1 (8.30%)

*binocular BCVA was used whenever available, else, the BCVA of the better eye was used

**all patients carried pathogenic or likely pathogenic variants

### Symptoms and impacts

**Fig. 3:**
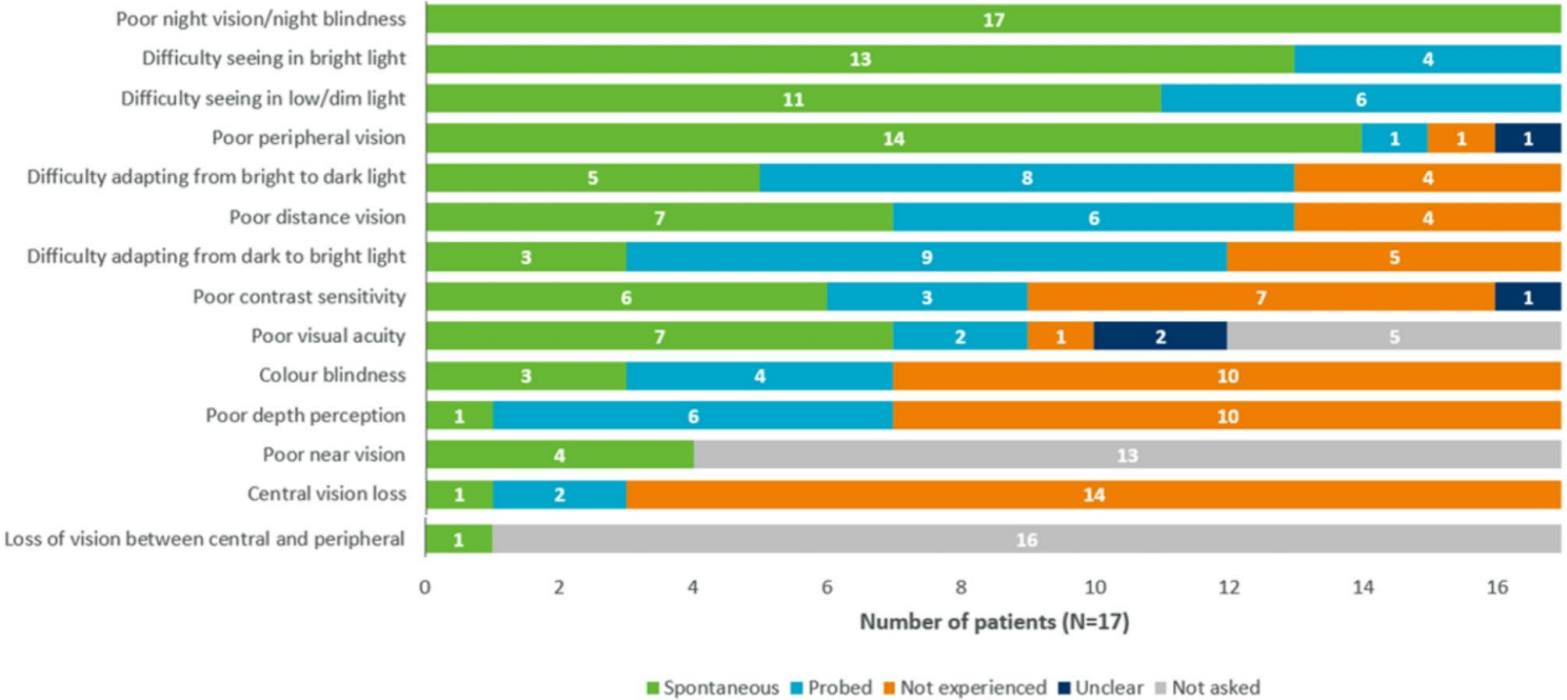
Symptoms reported by patients at the total sample

A total of 14 symptoms were reported by RP and CHM patients. The most frequently reported symptoms were poor night vision/night blindness (*n* = 17/17, 100%), difficulty seeing in bright light (*n* = 17/17, 100%), and difficulty seeing in low/dim light (*n* = 17/17, 100%) (Fig. 3). More than half of the participants in either condition reported difficulty adapting from bright to dark and vice versa, poor peripheral vision, poor contrast sensitivity, poor distance vision, and poor visual acuity (Table 5).

The terms used to describe the most common symptoms are given in Table 4.

**Table 4:** Terms used to describe the most common symptoms

Term used	Example quotes
Poor night vision/night blindness	
Can’t see/prevents from seeing [*n* = 6]	*“Basically*,* I say that it is a disease that prevents me from seeing at night.”* (1005-CHM-MIL*) *“While me*,* I can’t see at night.”* (2005-RP-SEV*)
Difficulty seeing/see poorly/see badly [*n* = 6]	*“I have never seen well at night*,* I don’t know when exactly it changed. I have always seen poorly at night…”* (2003-RP-MIL*) *“And I see very*,* very badly at night*,* which means that*,* uh*,* well*,* when it’s dark*,* I have a lot of trouble moving around”* (2007-RP-MOD)
Night vision [*n* = 2]	*“…the first signs that I really noticed myself were really in my vision in the dark.”* (2011-RP-MOD)
Other descriptions: difficulty at night (2008-RP-MOD*), bothered (1003-CHM-MIL*), uncomfortable (1001-CHM-SEV*)	
Difficulty seeing in bright light	
Blinded/dazzled [*n* = 11]	*“There are days when*,* if someone is standing in a place where there is lighting behind them*,* I can’t see them.”* (2003-RP-MIL*) *“Because*,* since the sun*,* well*,* is too strong*,* it hurts my eyes. And I can’t actually see where I’m going anymore.”* (2007-RP-MOD*)
Can’t see/ difficulty seeing [*n* = 5]	*“Yes*,* yes*,* absolutely*,* when there is a lot of sun. I don’t go skiing any more*,* for example*,* where the white reflects the sun*,* it’s not possible.”* (2011-RP-MOD*) *“I get blinded by glare*,* much more than other people are… So it can be headlamps*,* LED light bulbs*,* and all that.”* (1001-CHM-SEV*)
Difficulty with glare/ reflections [*n* = 4]	*Yes. I am photosensitive so*,* when it is sunny*,* it’s very uncomfortable and I have to put on sunglasses* (2007-RP-MOD)
Sensitive to light/photosensitivity [*n* = 3]	*But*,* during the summer*,* if there is too much*,* too much sun*,* I can’t go out without my sunglasses. That doesn’t really hurt my eyes too much. Now*,* it’s true that the sun hurts my eyes*,* you know. It really makes my eyes water* (1005-CHM-MIL*)
Hurts [*n* = 3]	*“Because*,* since the sun*,* well*,* is too strong*,* it hurts my eyes. And I can’t actually see where I’m going anymore.”* (2007-RP-MOD*)
Other descriptions: Sensitive to contrast (1004-CHM-MIL), disabling (1001-CHM-SEV*), incapacitating (1001-CHM-SEV*)	
Difficulty seeing in low/dim light	
Can see a little bit/see less/don’t see well [*n* = 6]	“*So*,* the very first time*,* I had gone for a walk with my family in a cave. And I realised that I could see much less well than the last time. And I started to question myself*,* and the further we went*,* well there’s not much light in these caves*,* and I could see less and less.”* (2004-RP-MIL*) *“In fact*,* at dusk*,* that’s when I don’t see as well*,* and at sunrise”* (1005-CHM-MIL*)
Becomes more difficult [*n* = 3]	*“An activity can start in daylight*,* but if it’s in a place where there is very little light*,* it may become difficult”* (2002-RP-MIL*) *“From the moment that the sun starts to set*,* it becomes difficult. And it’s the most difficult when it is completely night”* (2007-RP-MOD*)
Unable to identify/recognize details/objects [*n* = 2]	*“If*,* for example*,* the light*,* I go into an office and the light is dim or low*,* I won’t see the chair to sit down… it prevents me from identifying objects”* (2005-RP-SEV*)
Can’t see [*n* = 2]	*“…if I go to a restaurant*,* the*,* the… it’s dim and*,* well*,* I can’t see anything. Actually*,* for me*,* uh*,* dim light*,* it’s the same as the night outside*,* you know?”* (2008-RP-MOD*)
Other descriptions: uncomfortable (1001-CHM-SEV)	
Poor peripheral vision	
Narrowing/ reduction of visual field [*n* = 12]	*“…what I noticed over time with age*,* in fact*,* is that it was really a narrowing of the visual field*,* uh… on the sides on the one hand*,* but also up high and… actually*,* horizontally but also vertically.”* (2010-RP-SEV*) *“…a reduced visual field*,* which makes me have to look at my feet. So*,* when I look at my feet*,* I don’t look at what’s in front of me*,* so*,* it was tough.”* (1004-CHM-MIL*)

**Other descriptions**: Peripheral vision (2001-RP-SEV*), difficulty seeing below nose (1001-CHM-SEV*)

*coding: patient number - condition (RP or CHM)– disease severity (MIL = mild, MOD = moderate, SEV = severe as defined in Fig. 2)

Table 4: ***Terms used to describe the most common symptoms [see end of text body]***.

Most symptoms were consistently reported by RP and CHM patients (Table 5), with 11 of the 14 symptoms reported by at least one patient with each condition. Exceptions were color blindness, central vision loss, and loss of vision between central and peripheral vision, which were only reported by RP patients.

Similarly, the most common symptoms were reported consistently by patients with different disease severity levels (Table 5). The less common symptoms, such as poor visual acuity, poor depth perception and color blindness, were more commonly reported by patients with greater disease severity.

**Table 5:** Symptoms according to condition and disease severity (Iftikhar grades)

Condition/Disease severity (Iftikhar grades)	Total (*n* = 17)		RP (*n* = 12)		CHM (*n* = 5)		Grade 2 (*n* = 4)		Grade 3 (*n* = 5)		Grade 4 & 5 (*n* = 8)	
Symptom	n	%	n	%	n	%	n	%	n	%	n	%
Poor night vision/night blindness	17	100	12	100	5	100	4	100	5	100	8	100
Difficulty seeing in bright light	17	100	12	100	5	100	4	100	5	100	8	100
Difficulty seeing in low/dim light	17	100	12	100	5	100	4	100	5	100	8	100
Poor peripheral vision	15	88.2	10	83.3	5	100	4	100	4	80.0	7	87.5
Difficulty adapting from bright to dark light	13	76.5	9	75.0	4	80.0	3	75.0	3	60.0	7	87.5
Poor distance vision	13	76.5	10	83.3	3	60.0	2	50.0	4	80.0	7	87.5
Difficulty adapting from dark to bright light	12	70.6	9	75.0	3	60.0	3	75.0	3	60.0	6	75.0
Poor contrast sensitivity	9	52.9	6	50.0	3	60.0	2	50.0	1	20.0	6	75.0
Poor visual acuity	9	52.9	6	50.0	3	60.0	1	25.0	1	20.0	7	87.5
Color blindness	7	41.2	7	58.3	0	0	0	0.0	1	20.0	6	75.0
Poor depth perception	7	41.2	4	33.3	3	60.0	1	25.0	0	0.0	6	75.0
Poor near vision	5	29.4	3	25.0	1	20.0	2	50.0	2	40.0	0	0
Central vision loss	3	17.6	3	25.0	0	0	1	25.0	0	0	2	25.0
Loss of vision between central and peripheral	1	5.9	1	8.3	0	0	0	0.0	0	0	1	12.5

Multiple answers were possible.

Symptoms not mentioned spontaneously, probed for, or with unclear answers were categorized as “not reported”

Nearly all patients reported what they considered the most bothersome symptom (*n* = 14/17, 82.4%), the most important symptom to improve with treatment (*n* = 15/17, 88.2%), and the most important symptom to stabilize or delay decline with treatment (*n* = 15/17, 88.2%) (Table 6). In all these ratings, participants mentioned poor peripheral vision and poor night blindness in first or second place. Poor night vision was also the most frequent reason (*n* = 11/17, 64.7%) for seeking help from a healthcare professional.

**Table 6:** Patient’s rating of symptoms (bothersome/important to improve/important to stabilize) by condition

Patients’ rating	Most bothersome symptom				Most important to improve with treatment				Most important to stabilize/delay decline with treatment			
Answers	RP (*n* = 11)		CHM (*n* = 3)		RP (*n* = 11)		CHM (*n* = 4)		RP (*n* = 11)		CHM (*n* = 4)	
Symptom	RP n	RP %	CHM n	CHM %	RP n	RP %	CHM n	CHM %	RP n	RP %	CHM n	CHM %
Poor night vision/night blindness	3	27.3	1	33.3	7	63.6			3	27.3	1	25.0
Difficulty seeing in bright light	1	9.1							1	9.1	1	25.0
Poor peripheral vision	5	45.5	1	33.3	3	27.3	2	50.0	3	27.3	3	75.0
Difficulty adapting from bright to dark light	1	9.1										
Poor distance vision	1	9.1			1	9.1						
Difficulty adapting from dark to bright light					1	9.1						
Poor contrast sensitivity			1	33.3			1	25.0				
Poor visual acuity	1	9.1					1	25.0				
Central vision loss									3	27.3		
Overall symptoms					1	9.1						
Overall disease progression									3	27.3		
General impaired vision	1	9.1										
Multiple answers were possible.												

The impact of disease symptoms on patients’ lives was significant. Table 7 shows how individual symptoms had an impact on a wide range of activities.

**Table 7:** Number of RP and CHM patients reporting symptoms to cause or exacerbate impacts

Impact Symptom	Ability to navigate	Use of digital screens	Physical functioning	Travel	Reading	Activities of daily living	Work/school	Social functioning	Emotional wellbeing
Central vision loss				1					
Color blindness	2					2			
Difficulty adapting from bright to dark light									
Difficulty adapting from dark to bright light			1						1
Difficulty seeing in bright light	1	5	2			2	1	5	
Difficulty seeing in low/dim light								**12**	
Poor contrast sensitivity			7		5	6	2		
Poor depth perception	4		8	1					1
Poor distance vision			2	1	1		2		
Poor near vision									
Poor night vision/night blindness	2		3	3			3	7	2
Poor peripheral vision	**10**	2	8	5		7	2	4	
Poor visual acuity		3		1		1			2

**Fig. 4:**
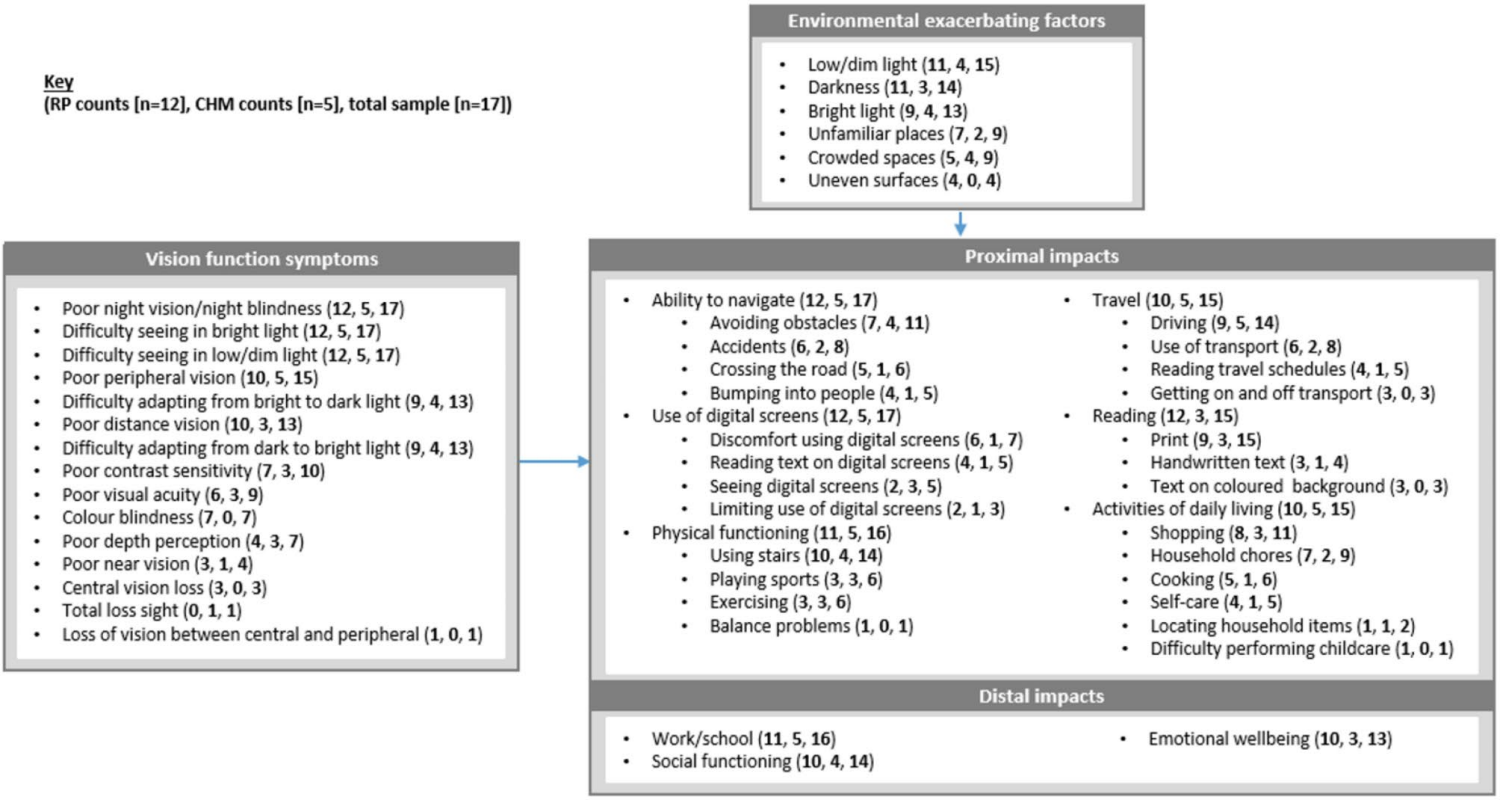
Conceptual model of RP and CHM symptom and impact concepts

For our conceptual model (Fig. 4), we grouped the impact concepts into proximal functional impacts on ADLs and distal impacts on wider quality of life (including work, social and emotional impacts).

The most notable impacts were on the ability to navigate (*n* = 17/17, 100%), use of digital screens (*n* = 17/17, 100%), physical functioning (*n* = 16/17, 94.1%), and work/school-related activities (*n* = 16/17, 94.1%). Environmental exacerbating factors were most often reported in connection with navigation. All participants (*n* = 17/17, 100%) reported that their ability to navigate was impacted by environmental conditions. Most often, they mentioned darkness (*n* = 9/17, 52.9%), unfamiliar places (*n* = 9/17, 52.9%), and crowded spaces (*n* = 9/17, 52.9%). Other environmental conditions exacerbated disease impacts on social functioning (*n* = 12/14, 85.7%) and reading (*n* = 5/15, 33.3%).

Health-related Quality of Life (HRQoL) impacts associated with emotional wellbeing were also identified (*n* = 13/17, 76.5%). Patients most frequently reported sadness/depression due to their eye condition (*n* = 6/13, 46.2%), followed by annoyance/frustration (*n* = 4/13, 30.8%), guilt/burden (*n* = 3/13, 23.1%), anxiety/stress (*n* = 2/13, 15.4%), fear (*n* = 2/13, 15.4%), anger (*n* = 2/13, 15.4%), powerlessness (*n* = 2/13, 15.4%), and embarrassment/self-consciousness (*n* = 2/13, 15.4%). All impacts on emotional well-being were mentioned spontaneously and not specifically probed upon during the interviews.

As for the symptoms, RP and CHM patients reported impacts consistently, with all domains of impact reported by each group (Table 8). Similarly, patients within each level of disease severity reported experiencing all domains of impact. Of note, participants with more severe disease generally reported experiencing more impacts.

**Table 8:** Proximal and distal impacts according to condition and disease severity

		Condition/ Iftikhar grade	Total (*n* = 17)		RP (*n* = 12)		CHM (*n* = 5)		Grade 2 (*n* = 4)		Grade 3 (*n* = 5)		Grade 4 & 5 (*n* = 8)	
		Impacts	n	%	n	%	n	%	n	%	n	%	n	%
Proximal	Navigation	Ability to navigate	17	100.0	12	100.0	5	100.0	4	100.0	5	100.0	8	100.0
		Avoiding obstacles	11	64.7	7	58.3	4	80.0	3	75.0	3	60.0	5	62.5
		Accidents	8	47.1	6	50.0	2	40.0	2	50.0	2	40.0	4	50.0
		Crossing the road	6	35.3	5	41.7	1	20.0	2	50.0	1	20.0	3	37.5
		Bumping into people	5	29.4	4	33.3	1	20.0	0	0.0	2	40.0	3	37.5
	Digital screen use	Use of digital screens	17	100.0	12	100.0	5	100.0	4	100.0	5	100.0	8	100.0
		Discomfort using digital devices	7	41.2	6	50.0	1	20.0	3	75.0	3	60.0	1	12.5
		Reading text on digital devices	5	29.4	4	33.3	1	20.0	0	0.0	2	40.0	3	37.5
		Difficulty seeing digital screens	5	29.4	2	16.7	3	60.0	1	25.0	0	0.0	4	50.0
		Limiting use of digital screens	3	17.6	2	16.7	1	20.0	0	0.0	2	40.0	1	12.5
	Travel	Travel	15	88.2	10	83.3	5	100.0	3	75.0	4	80.0	8	100.0
		Driving	14	82.4	9	75.0	5	100.0	3	75.0	4	80.0	7	87.5
		Use of transport	8	47.1	6	50.0	2	40.0	1	25.0	1	20.0	6	75.0
		Reading travel schedules	6	35.3	4	33.3	2	40.0	1	25.0	2	40.0	3	37.5
		Getting on and off transport	3	17.6	3	25.0	0	0.0	1	25.0	1	20.0	1	12.5
	Reading	Reading	15	88.2	12	100.0	3	60.0	4	100.0	4	80.0	7	87.5
		Print	12	70.6	9	75.0	3	60.0	3	75.0	3	60.0	6	75.0
		Handwritten text	3	17.6	3	25.0	0	0.0	1	25.0	0	0.0	2	25.0
		Text on colored background	4	23.5	3	25.0	1	20.0	2	50.0	1	20.0	1	12.5
	Activities of daily living	Activities of daily living	15	88.2	10	83.3	5	100.0	3	75.0	4	80.0	8	100.0
		Shopping	11	64.7	8	66.7	3	60.0	2	50.0	1	20.0	8	100.0
		Household chores	9	52.9	7	58.3	2	40.0	2	50.0	1	20.0	6	75.0
		Cooking	6	35.3	5	41.7	1	20.0	1	25.0	2	40.0	3	37.5
		Personal care/putting on make-up	5	29.4	4	33.3	1	20.0	1	25.0	1	20.0	3	37.5
		Locating household items	1	5.9	1	8.3	0	0.0	0	0.0	0	0.0	1	12.5
		Difficulty performing childcare	2	11.8	1	8.3	1	20.0	1	25.0	0	0.0	1	12.5
	Physical functioning	Physical functioning	16	94.1	11	91.7	5	100.0	3	75.0	5	100.0	8	100.0
		Using stairs	14	82.4	10	83.3	4	80.0	2	50.0	5	100.0	7	87.5
		Sports	6	35.3	3	25.0	3	60.0	2	50.0	0	0.0	4	50.0
		Exercise	3	17.6	3	25.0	0	0.0	1	25.0	0	0.0	2	25.0
		Balance problems	4	23.5	1	8.3	3	60.0	1	25.0	0	0.0	3	37.5
Distal		Work/school	16	94.1	11	91.7	5	100.0	4	100.0	4	80.0	8	100.0
		Social functioning	14	82.4	11	91.7	5	100.0	2	50.0	4	80.0	8	100.0
		Emotional wellbeing	13	76.5	10	83.3	4	80.0	4	100.0	2	40.0	7	87.5

Additional quantitative analyses were conducted in which the presence of individual symptoms was analyzed according to the presence or absence of a range of clinical parameters. We found that (1) patients typically experienced many symptoms at the same time, i.e., if one symptom was present, further symptoms were also present. (2) Patients who reported color blindness (all had RP) had a lower BCVA score, a narrower ellipsoid zone, a higher Iftikhar cumulative score, and a lower GVF score than those who reported color blindness. More details on the quantitative analysis of symptoms by clinical variables are available in the Supplement (Figs. 5, 6, 7, 8 and 9).

### Conceptual saturation analysis

For the RP sample (*n* = 12), all symptom concepts were mentioned spontaneously in the first three sets of patient interviews, with most symptoms mentioned spontaneously in the first set of interviews (85.7%) (Table 12 - supplement). This suggests that further interviews in the RP sample would be unlikely to yield any new symptom concepts.

In the overall sample (*n* = 17), all symptom concepts were mentioned spontaneously in the first two sets of patient interviews, with most symptoms mentioned spontaneously in the first set of interviews (85.7%) (Table 13 - supplement). Of note, while the first three sets of interviews were conducted with RP patients, the last set of interviews was conducted with CHM patients only. No CHM patients reported color blindness, central vision loss or loss of vision between central and peripheral field of view.

Conceptual saturation was also assessed for the impact concepts at the overall sample and individually for RP. In the RP sample (*n* = 12), all impact concept domains were mentioned spontaneously in the first set of patient interviews (Table 14 - supplement). Similarly, most subconcepts for proximal impacts were mentioned spontaneously in the first two sets of interviews (84.0%). Exceptions were crossing the road (ability to navigate domain), reading text on a colored background (reading domain), and balance problems (physical functioning domain), which were mentioned spontaneously only in the last set of interviews and by one patient each.

For the overall sample (*n* = 17), all impact concept domains (100%) were spontaneously elicited in the first set of patient interviews (Table 15 - supplement). Similarly, all subconcepts for the proximal impacts were mentioned spontaneously in the first three sets of patient interviews, with most of these subconcepts mentioned spontaneously in the first set of interviews (64.0%).

The small sample size of CHM patients (*n* = 5) did not allow assessing conceptual saturation in this population.

## Discussion

In this study, we conducted qualitative concept elicitation (CE) interviews with RP and CHM patients to explore their disease experience. We identified symptoms, along with the impacts of these symptoms on ADL and HRQoL, as well as exacerbating environmental factors. From this, we developed a conceptual model suitable for informing recommendations for the future development of PROs and PerfOs. Additionally, we assessed clinical and imaging parameters to understand how disease severity is linked to the participants’ disease experience.

### Symptoms and impacts

Overall, 14 symptoms were identified. Of these, 11 were relevant to both RP and CHM patients. All participants, irrespective of condition, reported poor night vision/night blindness, difficulty seeing in bright light and difficulty seeing in low/dim light.

All symptoms were reported by similar proportions of patients from each condition, with the exception of color blindness, central vision loss, and loss of vision between central and peripheral, which were only reported for RP. However, this finding may be due to the small CHM sample size. Nevertheless, symptoms showed a high similarity between the two populations, despite the different disease etiologies. This is in line with the literature, which indicates that the symptoms of both diseases are so similar that careful ophthalmoscopy or even genetic testing is required for unequivocal differential diagnosis [15–20, 22].

The symptoms reported correspond to the existing clinical descriptions of RP and CHM. These point out night blindness at the beginning of the disease, followed by progressive loss of the peripheral visual field in daylight, eventually leading to blindness after several decades and, in some patients, difficulties adjusting to changing levels of light and difficulty seeing in poor contrast [3, 4].

Due to the similarity of symptoms observed in our study and in the literature, most of the discussion will focus on the analysis of the total study population, i.e., RP and CHM combined. Comparisons between conditions and disease severity should be interpreted as exploratory due to the small group sizes.

When looking at the symptoms reported across different disease severities, the most common symptoms were present in similar proportions across disease severities, whereas the less common symptoms, such as color blindness, poor depth perception, poor near vision, and central vision loss, were more often reported by participants with more severe disease.

Moreover, we gained insight into which symptoms were most important to patients. The majority of patients described poor peripheral vision and poor night vision/night blindness as either the most bothersome, most important to stabilize, or as the most important to improve with treatment.

Nearly all patients reported disease impact on the abilities to navigate (100%), using digital screens (100%), physical functioning (94%), travel (88%), reading (88%), ADLs (88%), work- or school-related activities (94%), social functioning (82%) and emotional well-being (77%). Importantly, the impact of symptoms was typically exacerbated through environmental factors, especially for navigating (100%), social functioning (85.7%) and reading (33%). The most commonly mentioned exacerbating factors were lighting conditions (bright, dark, low/dim) as well as unfamiliar places and crowded spaces. The fact that over three quarters of participants reported impacts on their social functioning and emotional well-being shows that retinal diseases have a strong impact on HRQoL. Therefore, assessments of HRQoL should form an essential part of future studies.

All disease impact domains were relevant in both conditions, and participants with more severe disease experienced twice as many impacts as participants with less severe disease.

Our findings are comparable to those of Green et al., who performed a concept elicitation study in RLBP1-related RP patients [23], while no comparable study on CHM is known to us. In Green’s study, the most frequently reported symptoms were night blindness (100%), reduced light/dark adaptation (100%), reduced vision in bright light (81%), loss of visual acuity (71%), color blindness (62%), and loss of peripheral vision (62%).

The most frequently reported impacts concerned ADL (100%), emotions (90%), reading (81%), driving (71%), chores and cleaning (62%), navigation (52%), sports/physical activity (52%), and walking into objects (48%). Impacts were reported to be exacerbated by lighting conditions, unfamiliar environments and weather conditions. As in our study, they observed that participants with more severe disease appeared to have a more severe experience of certain symptoms and impacts than did participants with milder disease.

### Concept saturation

Achieving conceptual saturation is important for ensuring an adequate sample size and is determined based on spontaneous reports of concepts only [9, 10]. In our study, concept saturation was achieved in RP patients (*n* = 12) for nearly all symptoms but not in CHM patients (*n* = 5). A total of 12 participants have also been described in the literature as the minimum number needed to achieve conceptual saturation in a relatively homogeneous population [9, 10]. The reason for the small number of participants with CHM lies in the rareness of the disease, which led to slow recruitment so that at the time of study closure, only 5 CHM patients had been recruited. Likewise, the subgroups with different disease severities were too small to reach saturation. Therefore, any comparison of conditions or disease severities made in this paper is exploratory only and should be interpreted with caution.

### Strengths and limitations

This is the first concept elicitation study in RP and CHM patients that describes the reported concepts in relation to disease severity graded with a disease-specific assessment.

Its main limitations are the relatively small study population and the fact that the study was conducted at a single site.

The fact that we achieved concept saturation only for RP but not for CHM mainly reflects the rareness of CHM, with its inherent difficulties in recruitment. Since this was already expected at the time of study initiation, the objectives of the study took into account that no exhaustive information about CHM was to be expected. Even though concepts relevant in CHM may not have been fully explored, this is the first time that concept elicitation interviews in CHM are published.

Likewise, the population was too small to achieve concept saturation for the individual disease severities. Nevertheless, we used quota for disease severities during recruitment to ensure that all disease severities would be represented in similar proportions.

The small sample size also precluded further analysis, e.g., correlating the patient experience (most bothersome) and treatment expectations (most important to stabilize / most important to improve with treatment) with disease severity.

Of note, we determined disease severity in RP patients as well as in CHM patients with a classification system originally developed for RP. Based on the similarities in clinical presentation and the lack of a comparable classification for CHM, this seemed to be the best option available.

Another limitation is that only participants with a BCVA of ≥ 55 ETDRS letter equivalent in one or both eyes were eligible for this study. Considering that both target conditions may eventually lead to blindness, this is an important limitation because our findings may not be generalizable to the most advanced disease stages. Finally, the majority of participants (*n* = 15) lived in an urban area, so our results may not have sufficiently covered impacts specific to rural living. The reason for this may be inherent to conducting a single-center study at an urban study site. However, the urban study population may also be a result of people with visual impairment moving from the countryside to the city since the urban environment is more easily accessible to them than a rural environment.

The greatest achievement of this study is that it generated results suitable for informing the development of future PROs and PerfOs targeted at both RP and CHM patients. This could be the first step to cover the unmet need for clinical outcome assessments (COAs) measuring symptoms that better reflect patients’ experience and needs [6].

## Conclusion

The patient interviews provided insight into the patient experience of RP and CHM to develop a combined conceptual model of the RP and CHM disease experience. The most common symptoms, i.e., poor night vision/night blindness, difficulty seeing in bright light and difficulty seeing in low/dim light, were reported by all participants, independent of condition and disease severity.

Our findings are suitable to inform recommendations for the development of patient-reported outcome (PRO) and performance outcome (PerfO) measures needed to evaluate novel therapies for both RP and CHM.

## Electronic supplementary material

Below is the link to the electronic supplementary material.

Supplementary Material 1

## Data Availability

Qualified researchers may request access to individual patient-level data by contacting the corresponding author.

## Abbreviations

ADL: Activities of daily living
BCVA: Best corrected visual acuity
CE: Concept elicitation
CHM: Choroideremia
CHNO: Centre hospitalier national d’ophtalmologie
COA: Clinical outcome assessments
ETDRS: Early treatment diabetic retinopathy study
GVF: Goldmann visual field
HRQOL: Health-related quality of life
IRDs: Rare inherited retinal diseases
OCT: Optical coherence tomography
PerfO: Performance outcomes
PRO: Patient-reported outcomes
RP: Retinitis pigmentosa

## References

[1] Hamel C. Retinitis pigmentosa. Orphanet J Rare Dis. 2006;1:40. 17032466 10.1186/1750-1172-1-40 PMC1621055

[2] Khan KN, Islam F, Moore AT, Michaelides M. Clinical and genetic features of choroideremia in childhood. Ophthalmology. 2016;123:2158–65. 27506488 10.1016/j.ophtha.2016.06.051

[3] Prem Senthil M, Khadka J, Pesudovs K. Seeing through their eyes: lived experiences of people with retinitis pigmentosa. Eye. 2017;31:741–8. 28085147 10.1038/eye.2016.315 PMC5437327

[4] Sankila E-M, Tolvanen R, van den Hurk JAJM, Cremers FPM, de la Chapelle A. Aberrant splicing of the CHM gene is a significant cause of choroideremia. Nat Genet. 1992;1:109–13. 1302003 10.1038/ng0592-109

[5] Braithwaite T, Calvert M, Gray A, Pesudovs K, Denniston AK. The use of patient-reported outcome research in modern ophthalmology: impact on clinical trials and routine clinical practice. Patient Relat Outcome Meas. 2019;10:9–24. 30774489 10.2147/PROM.S162802 PMC6352858

[6] Thompson DA, Iannaccone A, Ali RR et al. (2020) Advancing Clinical Trials for Inherited Retinal Diseases: Recommendations from the Second Monaciano Symposium. Transl Vis Sci Technol 9:2. 10.1167/tvst.9.7.2 PMC7414644 32832209

[7] FDA. (2022) Patient-Focused Drug Development: Methods to Identify What Is Important to Patients Guidance for Industry, Food and Drug Administration Staff, and Other Stakeholders. In Services USDoHaH, editor.

[8] Banhazi J, Williamson N, Bradley H, et al. PRO131 WHAT DO WE KNOW ABOUT PATIENT’S AND CAREGIVERS’ EXPERIENCE WHEN LIVING WITH THE HEREDITARY RETINAL CONDITION RETINITIS PIGMENTOSA? Value Health. 2019;22:S865.

[9] Francis JJ, Johnston M, Robertson C, Glidewell L, Entwistle V, Eccles MP, Grimshaw JM. What is an adequate sample size? Operationalising data saturation for theory-based interview studies. Psychol Health. 2010;25:1229–45. 20204937 10.1080/08870440903194015

[10] Guest G, Bunce A, Johnson L. How many interviews are enough? An experiment with data saturation and variability. Field Methods. 2006;18:59–82.

[11] Fereday J, Muir-Cochrane E. Demonstrating rigor using thematic analysis: A hybrid approach of inductive and deductive coding and theme development. Int J Qualitative Methods. 2006;5:80–92.

[12] Boyatzis RE. Transforming qualitative information: thematic analysis and code development. sage; 1998.

[13] Sandelowski M. Real qualitative researchers do not count: the use of numbers in qualitative research. Res Nurs Health. 2001;24:230–40. 11526621 10.1002/nur.1025

[14] Iftikhar M, Lemus M, Usmani B, Campochiaro PA, Sahel JA, Scholl HPN, Shah SMA. Classification of disease severity in retinitis pigmentosa. Br J Ophthalmol. 2019;103:1595. 30705041 10.1136/bjophthalmol-2018-313669

[15] Guo H, Li J, Gao F, Li J, Wu X, Liu Q. Whole-exome sequencing reveals a novel CHM gene mutation in a family with choroideremia initially diagnosed as retinitis pigmentosa. BMC Ophthalmol. 2015;15:85. 26216097 10.1186/s12886-015-0081-4 PMC4517409

[16] Kim JH, Han JW, Choi EW, Bang JH, Shin HJ, Jang MA, Lee JY, Choi JN, Chang HS, Park TK. Clinical manifestations and genetic analysis of 5 Korean choroideremia patients initially diagnosed with retinitis pigmentosa. J Korean Med Sci. 2022;37:e5. 35040292 10.3346/jkms.2022.37.e5 PMC8763878

[17] Lam BL, Davis JL, Gregori NZ. Choroideremia gene therapy. Int Ophthalmol Clin. 2021;61:185–93. 34584056 10.1097/IIO.0000000000000385 PMC8478312

[18] Nanda A, Salvetti AP, de la Martinez-Fernandez C, MacLaren RE. Misdiagnosis of X-linked retinitis pigmentosa in a choroideremia patient with heavily pigmented fundi. Ophthalmic Genet. 2018;39:380–3. 29377744 10.1080/13816810.2018.1430242

[19] Yang J, Wang LN, Yu RG, Hu LY, Gong X, Chen L, Hu BJ, Li XR, Li ZQ. Multimodal imaging of the carriers of choroideremia and X-linked retinitis pigmentosa. Int J Ophthalmol. 2018;11:1721–5. 30364247 10.18240/ijo.2018.10.23 PMC6192969

[20] Zinkernagel MS, MacLaren RE. Recent advances and future prospects in choroideremia. Clin Ophthalmol (Auckland NZ). 2015;9:2195–200. 10.2147/OPTH.S65732 PMC4664510 26648685

[21] Boeije H. A purposeful approach to the constant comparative method in the analysis of qualitative interviews. Qual Quantity. 2002;36:391–409.

[22] Nouraeinejad A. How to make a differential diagnosis between choroideremia and retinitis pigmentosa. Pan-American J Ophthalmol. 2022;4:8–8.

[23] Green J, Tolley C, Bentley S, et al. Qualitative interviews to better understand the patient experience and evaluate patient-Reported outcomes (PRO) in RLBP1 retinitis pigmentosa (RLBP1 RP). Adv Therapy. 2020;37:2884–901. 10.1007/s12325-020-01275-4 PMC7467452 32372289

