# Supplementary material for: Patient experience in retinitis pigmentosa and Choroideremia– a concept elicitation study in 17 patients based on qualitative interviews
Source: Orphanet J Rare Dis. 2025 Aug 11;20:418. doi: 10.1186/s13023-025-03713-4 (PMC12337473; doi:10.1186/s13023-025-03713-4)
Supplement: Supplementary file 1 — Supplementary Material 1 [file 13023_2025_3713_MOESM1_ESM.docx]

**Patient experience in Retinitis Pigmentosa and Choroideremia – a concept elicitation study in 17 patients based on qualitative interviews -SUPPLEMENT**

**Part 1 (pages 1-5): Conduct of Interviews - core symptoms, concept and related probes**

**Part 2 (pages 6-11): Conceptual saturation tables**

**Part 3 (pages 12-16): Quantitative analysis of symptoms by clinical variables**

**Part 1: Conduct of Interviews - core symptoms and concept and related probes**

Table 9: Probes for symptom/vision problems mentioned

| Description | - How would you describe this symptom? How does it make you feel? - Tell me about any additional words you use to describe this symptom. |
| --- | --- |
| Frequency* | - How often do you experience this symptom? - Does the frequency vary/has it changed over time? Please tell me about this. - If so, in which situations does the frequency vary (e.g., daytime vs night-time, lighting conditions, the environment you are in and its familiarity)? |
| Duration* | - How long does this symptom typically last for? - Does the duration vary/has it changed over time? Tell me about this |
| Severity | - When you have this symptom, how bad or severe is it usually? At its worst? - How would you rate this symptom on a scale from 0-10, where 0 is no symptom and 10 is extremely severe? - Does the severity vary? Please tell me about this. - If so, in what situations does the severity vary (e.g., daytime vs night-time, lighting conditions, the environment you are in?) - Has the severity changed over time? Please tell me about this |
| Impact/situation | - How does this symptom affect your life? Tell me about that - Are there certain situations or environments when this symptom is most impactful (e.g., daytime vs night-time, lighting condition, the environment you are in and its familiarity)? - Are there things you are not able to do or that are more difficult because of this symptom? - Is there anything you avoid doing because of this symptom? |
| Behavior | - Tell me about anything you do or use to cope with/manage the effects of this symptom? |
| Not all of these symptom characteristics were asked of all patients due to the semistructured nature of the interviews along with time constraints  *only asked for symptoms that were not constant (e.g., difficulty adapting to light changes, poor contrast sensitivity, and visual acuity) | |

Table 10: Examples of impact concepts and environmental conditions probed on in the interviews

| Impact domain* | Impact probes | Environmental conditions |
| --- | --- | --- |
| Physical functioning | - Mobility - Walking - Climbing stairs | - Busy environments/crowds - Lighting conditions - Time of day - Unfamiliar environment - Ambient/background noise |
| Navigation | - Avoiding obstacles - Crossing the road - Accidents | - Crowded spaces - Uneven surfaces - Unfamiliar places |
| Activities of daily living – in the home | - Household chores - Cooking or preparing food - Self-care activities (e.g., bathing, dressing, grooming) - Leisure activities (e.g., watching TV, reading a book, using a computer/tablet/phone, gardening) | - Lighting conditions - Time of day |
| Activities of daily living – outside the home | - Shopping (groceries and other items) - Leisure activities (e.g., go to restaurants, cinema, theatre, sport events) - Sports or exercise (e.g., running, walking, hiking) | - Lighting conditions - Specific environments - Day-to-day variation |
| Travel | - Use of transport (e.g., bus, train, bike, car) - Getting on and off transport - Buying a train ticket - Reading signs (e.g., timetables, finding platform) - Drive a car | - Lighting conditions - Specific environments - Day-to-day variation |
| Reading | - Print (e.g., in books, newspapers, bills, paperwork, street signs) - Small print (e.g., menu in a restaurant, medicine packaging or instructions, food labels) - Handwritten information/letters - Text on colored background - Moving text while still (e.g., train schedules on a digital board) - Static text on a moving object | - Lighting conditions - Specific environments |
| Use of electronic devices | - Reading text on digital device - Use of social media - Sending text messages | - Lighting conditions - Specific environments - Day-to-day variation |
| Work/school | - Getting to and from work/school - Navigating workplace/school - Carrying out assigned work/school activities - Use of equipment at work/school - Interacting with fellow students/colleagues or customers - Financial impacts | N/A |
| Social life/social activities | - Participation in social activities - Relationship with friends and family | N/A |
| Emotional wellbeing | N/A | N/A |

* Note: During analysis and for the purposes of reporting, some of the impacts were categorized under a different impact domain than how they were presented in the guide. This decision was based on how patients described these impacts. For instance, leisure activities outside the home were largely moved to social functioning, as patients tended to describe these activities as an impact on their ability to engage in social activities.

Table 11: Examples for code assignment from the original transcript

| **Original transcript** | **Codes assigned** |
| --- | --- |
| INTERVIEWER: […] So, can you tell me about the first changes, eh, if we go back, that you noticed in your vision, or the first problems that led you to consult a physician?  PARTICIPANT:  Okay. So, the first thing that really happened was night vision, uh, in adolescence. I realised at that time, when I went out with… groups of friends and uh… I realised that there was something that I couldn’t see… that I could see at night, you know. So, I always had a torch, I was careful or I let a friend that I was with know… who would take my hand or my arm, in short. It was really at that point that I thought to myself that it wasn’t normal.  INTERVIEWER:   Okay.  PARTICIPANT: That said, uh, I wasn’t diagnosed at that point, I was diagnosed later. But, I was suspicious at that time that there was something wrong.  INTERVIEWER:  What caused you to be diagnosed later?  PARTICIPANT:  So, I had, uh... I had seen an ophthalmologist, actually, I had already seen an adolescent ophthalmologist, but they had not diagnosed me. Uh...there you have it. Ultimately, they didn’t see it. And later I saw another ophthalmologist, I was 28 years old, because I… visually, I was… I couldn’t see well, I got tired quickly when I was working as well. And I thought to myself, “there really is something wrong.” And I saw an ophthalmologist who diagnosed the retinitis. | CE-RP-Symptom-Poor night vision/night blindness-Coping method-Torch  CE-RP-Symptom-Poor night vision/night blindness-S  CE-RP-Symptoms-Onset of symptoms-Poor night vision/night blindness-Adolescence  CE-RP-Symptoms-Reason for going to HCP-Poor night vision/night blindness |
| INTERVIEWER: Okay. When you told me that, visually you couldn’t see well, can you describe what you meant by that?  PARTICIPANT: I noticed that my distance vision, uh, I wasn’t… I couldn’t see some things that others saw or I didn’t see some details, well, there were things that I couldn’t see, you know. It was more for distance vision than for near vision at that point. | CE-RP-Symptom-Poor distance vision-S |
| INTERVIEWER: […] Thank you. Is your retinitis pigmentosa accompanied by difficulties adjusting to changes in lighting? I will start with the transition from the darkness into the light? Do you have any difficulty...  PARTICIPANT:  Oh yes, yes.  INTERVIEWER ... does it take you more time than others…  PARTICIPANT:  Oh yes, ah yes.  INTERVIEWER:…  ... to adjust your...  PARTICIPANT:  Yes.  INTERVIEWER:   Yes? Okay.  PARTICIPANT:  Oh yes, yes, yes. Yeah. Yes, yes, yes.  INTERVIEWER:  Would you have an example to give me?  PARTICIPANT:  Uh, from dark to light. Well, for example, I do a lot of hiking, so. When I go from… actually, this summer, for example, it happened to me several times, eh. When I go from a wooded area to a place, specifically, eh… into a clearing that is drenched in sunlight, well, I will advance carefully, you know. | CE-RP-Symptom-Difficulty adapting from dark to bright light-Description-Hiking from wooded to sunny area  CE-RP-Symptom-Difficulty adapting from dark to bright light - P |
| INTERVIEWER:   How often do you experience this symptom? Does it happen to you often?  PARTICIPANT:     Oh! all the time! Yes, yes, all the time. As soon as I go from… as soon as I go from… from two places where the light varies, I really, uh, in that direction, I really, uh, how should I say, yes, take my time. | CE-RP-Symptom-Difficulty adapting from dark to bright light-Frequency-All the time |
| INTERVIEWER:   Okay and in the other direction then? From brightness to darkness?  PARTICIPANT:   Well, in the other direction, it’s...Ah, there I take the time to stop, eh. There, I take the time to stop and I can tell you that it therefore takes, one minute, it’s maybe… actually, it takes one minute for sure. For sure.  INTERVIEWER:  Okay.  PARTICIPANT:  And there are places where I… where I will stop, you know. If I don’t have help, I stop.  INTERVIEWER:  And does that happen to you often? Every day or less often?  PARTICIPANT:  Listen, uh, every time that I have to go, uh, well, it happens pretty often really, eh. When I go from a place, uh, I don’t want to say that it’s several times a day, but it happens pretty regularly. | CE-RP-Symptom-Difficulty adapting from bright to dark light-Duration-1 minute  CE-RP-Symptom-Difficulty adapting from bright to dark light-Frequency-Several times a day  CE-RP-Symptom-Difficulty adapting from bright to dark light-P |
| Code assignment: S= spontaneous; P= prompted | |

**Part 2: Conceptual saturation tables**

Table 12: Conceptual saturation analysis for symptom concepts at the RP sample

| Concept | Group 1 (n=3) | | | Group 2 (n=3) | | | Group 3 (n=3) | | | Group 4 (n=3) | | |
| --- | --- | --- | --- | --- | --- | --- | --- | --- | --- | --- | --- | --- |
|  | 2001-RP-SEV* | 2002-RP-MIL* | 2003-RP-MIL* | 2004-RP-MIL* | 2005-RP-SEV* | 2006-RP-MIL* | 2007-RP-MOD* | 2008-RP-MOD* | 2009-RP-MOD* | 2010-RP-SEV* | 2011-RP-MOD* | 2012-RP-SEV* |
| Chronological order | 1 | 2 | 3 | 4 | 5 | 6 | 7 | 8 | 9 | 10 | 11 | 12 |
| Poor night vision/ night blindness | **S** | S | S | S | S | S | S | S | S | S | S | S |
| Difficulty seeing in  bright light | P | **S** | S | P | S | S | S | S | P | S | S | S |
| Difficulty seeing in  low/dim light | P | **S** | S | S | P | S | S | S | S | P | P | P |
| Poor peripheral vision | **S** | S | S | NE | S | S | P | U | S | S | S | S |
| Difficulty adapting from bright to dark light | P | P | **S** | S | S | NE | S | NE | NE | P | P | P |
| Poor distance vision | **S** | S | P | P | S | S | S | P | P | S | NE | NE |
| Difficulty adapting from  dark to bright light | NE | P | **S** | NE | NE | P | P | P | P | P | P | S |
| Poor contrast sensitivity | U | **S** | P | NE | S | NE | P | S | NE | S | NE | NE |
| Poor visual acuity | **S** | U | X | X | X | S | S | S | X | P | X | S |
| Color blindness | P | NE | NE | NE | **S** | NE | S | P | P | S | NE | P |
| Poor depth perception | **S** | NE | NE | NE | P | NE | P | NE | NE | NE | NE | P |
| Poor near vision | X | X | **S** | X | X | X | X | X | S | X | S | X |
| Central vision loss | NE | NE | **S** | NE | NE | NE | P | NE | NE | P | NE | NE |
| Loss of vision between central and peripheral | X | X | X | X | X | X | X | **S** | X | X | X | X |
| Note. S = spontaneous, P = probed, NE = not experienced and U = unclear. X = not discussed.  *coding: patient number - condition (RP or CHM) – disease severity (MIL= mild, MOD=moderate, SEV=severe as defined in Figure 1) Bold large font represents the first time a concept was mentioned. | | | | | | | | | | | | |

Table 13: Conceptual saturation analyses for symptom concepts at total sample

| Concept | Group 1 (n=4) | | | | Group 2 (n=4) | | | | Group 3 (n=4) | | | | Group 4 (n=5) | | | | |
| --- | --- | --- | --- | --- | --- | --- | --- | --- | --- | --- | --- | --- | --- | --- | --- | --- | --- |
|  | 2001-RP-SEV* | 2002-RP-MIL* | 2003-RP-MIL* | 2004-RP-MIL* | 2005-RP-SEV* | 2006-RP-MIL* | 2007-RP-MOD* | 2008-RP-MOD* | 2009-RP-MOD* | 2010-RP-SEV* | 2011-RP-MOD* | 2012-RP-SEV* | 1001-CHM-SEV* | 1002-CHM-SEV* | 1003-CHM-MIL* | 1004-CHM-MIL* | 1005-CHM-MIL* |
| Chronological order | 1 | 2 | 3 | 4 | 5 | 6 | 7 | 8 | 9 | 10 | 11 | 12 | 13 | 14 | 15 | 16 | 17 |
| Poor night vision/ night blindness | **S** | S | S | S | S | S | S | S | S | S | S | S | S | S | S | S | S |
| Difficulty seeing in  bright light | P | **S** | S | P | S | S | S | S | P | S | S | S | P | S | S | S | S |
| Difficulty seeing in  low/dim light | P | **S** | S | S | P | S | S | S | S | P | P | P | S | P | S | S | S |
| Poor peripheral vision | **S** | S | S | NE | S | S | P | U | S | S | S | S | S | S | S | S | S |
| Difficulty adapting from  bright to dark light | P | P | **S** | S | S | NE | S | NE | NE | P | P | P | P | P | NE | P | S |
| Poor distance vision | **S** | S | P | P | S | S | S | P | P | S | NE | NE | P | S | P | NE | NE |
| Difficulty adapting from  dark to bright light | NE | P | **S** | NE | NE | P | P | P | P | P | P | S | S | P | NE | P | NE |
| Poor contrast sensitivity | U | **S** | P | NE | S | NE | P | S | NE | S | NE | NE | P | S | NE | S | NE |
| Poor visual acuity | **S** | U | X | X | X | S | S | S | X | P | X | S | S | S | P | U | NE |
| Color blindness | P | NE | NE | NE | **S** | NE | S | P | P | S | NE | P | NE | NE | NE | NE | NE |
| Poor depth perception | **S** | NE | NE | NE | P | NE | P | NE | NE | NE | NE | P | P | P | NE | P | NE |
| Poor near vision | X | X | **S** | X | X | X | X | X | S | X | S | X | X | X | X | X | S |
| Central vision loss | NE | NE | **S** | NE | NE | NE | P | NE | NE | P | NE | NE | NE | NE | NE | NE | NE |
| Loss of vision between  central and peripheral | X | X | X | X | X | X | X | **S** | X | X | X | X | X | X | X | X | X |
| Note. S = spontaneous, P = probed, NE = not experienced and U = unclear. X = not discussed.  *coding: patient number - condition (RP or CHM) – disease severity (MIL= mild, MOD=moderate, SEV=severe as defined in Figure 1)  Bold large font represents the first time a concept was mentioned. | | | | | | | | | | | | | | | | | |

Table 14: Conceptual saturation analysis for impact concepts at the RP sample

| Concept | Group 1 (n=3) | | | Group 2 (n=3) | | | Group 3 (n=3) | | | Group 4 (n=3) | | |
| --- | --- | --- | --- | --- | --- | --- | --- | --- | --- | --- | --- | --- |
|  | RP-SEV-2001* | RP-MIL-2002* | RP-MIL-2003* | RP-MIL-2004* | RP-SEV-2005* | RP-MIL-2006 | RP-MOD-2007* | RP-MOD-2008* | RP-MOD-2009* | RP-SEV-2010* | RP-MOD-2011* | RP-SEV-2012* |
| Chronological order | 1 | 2 | 3 | 4 | 5 | 6 | 7 | 8 | 9 | 10 | 11 | 12 |
| Proximal impacts | | | | | | | | | | | | |
| Ability to navigate | **S** | S | S | S | S | S | S | S | S | S | P | P |
| Avoiding obstacles | **S** | S | S | X | X | X | P | P | S | S | X | X |
| Accidents | **S** | X | S | X | X | X | S | S | S | X | S | X |
| Crossing the road | X | X | P | X | X | X | P | P | X | X | P | **S** |
| Bumping into people | **S** | X | X | X | S | X | X | S | S | X | X | X |
| Use of digital screens | **S** | S | S | P | S | S | P | P | P | P | S | S |
| Discomfort using digital devices | **S** | S | S | X | X | S | X | X | S | X | S | X |
| Reading text on digital devices | X | X | X | **S** | X | X | S | X | S | S | X | X |
| Difficulty seeing digital screens | X | X | X | X | X | X | X | **S** | X | X | X | S |
| Limiting use of digital screens | X | **S** | X | X | X | X | X | X | X | X | X | S |
| Travel | **S** | S | S | NE | S | NE | S | S | S | S | S | S |
| Driving | **S** | S | S | X | X | X | S | S | S | S | S | S |
| Use of transport | **S** | S | X | X | S | NE | S | P | NE | X | NE | S |
| Reading travel schedules | X | X | X | X | **S** | S | S | X | X | X | S | X |
| Getting on and off transport | X | **S** | S | X | X | X | X | P | X | X | X | X |
| Reading | **S** | S | S | P | S | S | S | P | S | S | S | S |
| Print | **S** | X | S | S | S | X | S | P | S | S | S | X |
| Handwritten text | P | X | P | X | X | X | X | X | X | S | X | X |
| Text on colored background | X | P | P | X | X | X | X | X | X | **S** | X | X |
| Activities of daily living | **S** | S | P | P | S | NE | P | S | NE | P | S | S |
| Shopping | **S** | P | P | X | S | X | P | S | X | P | X | S |
| Household chores | X | P | S | X | P | X | P | S | X | P | X | S |
| Cooking | X | **S** | S | X | S | X | P | X | X | X | S | X |
| Personal care/putting on make-up | X | X | **S** | X | X | X | S | P | X | X | S | X |
| Locating household items | X | X | X | X | **S** | X | X | X | X | X | X | X |
| Difficulty performing childcare | X | X | **S** | X | X | X | X | X | X | X | X | X |
| Physical functioning | **S** | P | P | P | P | NE | S | S | P | P | P | S |
| Using stairs | **S** | S | X | P | S | X | S | S | P | S | P | S |
| Sports | X | X | X | X | **S** | X | S | X | X | X | X | S |
| Exercise | X | X | P | X | **S** | NE | X | X | NE | X | NE | S |
| Balance problems | X | X | X | X | X | X | X | X | X | X | X | **S** |
| Distal impacts | | | | | | | | | | | | |
| Work/school | **S** | S | S | NE | S | S | S | P | S | S | S | P |
| Social functioning | P | **S** | S | S | P | NE | S | S | S | P | NE | S |
| Emotional wellbeing | **S** | P | S | NE | P | P | S | S | NE | S | S | S |
| Note. S = spontaneous, P = probed, NE = not experienced and U = unclear. X = not discussed.  *coding: patient number – condition (RP or CHM) – disease severity (MIL= mild, MOD=moderate, SEV=severe as defined in Figure 1)  Bold large font represents the first time a concept was mentioned. | | | | | | | | | | | | |

Table 15: Conceptual saturation analysis for impact concepts at the total sample

| Concept | Group 1 (n=4) | | | | Group 2 (n=4) | | | | Group 3 (n=4) | | | | Group 5 (n=5) | | | | |
| --- | --- | --- | --- | --- | --- | --- | --- | --- | --- | --- | --- | --- | --- | --- | --- | --- | --- |
|  | 2001-RP-SEV* | 2002-RP-MIL* | 2003-RP-MIL* | 2004-RP-MIL* | 2005-RP-SEV* | 2006-RP-MIL* | 2007-RP-MOD* | 2008-RP-MOD* | 2009-RP-MOD* | 2010-RP-SEV* | 2011-RP-MOD* | 2012-RP-SEV* | 1001-CHM-SEV* | 1002-CHM-SEV* | 1003-CHM-MIL* | 1004-CHM-MIL* | 1005-CHM-MIL* |
| Chronological order | 1 | 2 | 3 | 4 | 5 | 6 | 7 | 8 | 9 | 10 | 11 | 12 | 13 | 14 | 15 | 16 | 17 |
| Proximal impacts | | | | | | | | | | | | | | | | | |
| Ability to navigate | **S** | S | S | S | S | S | S | S | S | S | P | P | S | S | S | S | S |
| Avoiding obstacles | **S** | S | S | X | X | X | P | P | S | S | X | X | S | X | S | S | S |
| Accidents | **S** | X | S | X | X | X | S | S | S | X | S | X | S | X | X | X | S |
| Crossing the road | X | X | P | X | X | X | P | P | X | X | P | **S** | X | X | X | X | P |
| Bumping into people | **S** | X | X | X | S | X | X | S | S | X | X | X | X | X | S | X | X |
| Use of digital screens | **S** | S | S | P | S | S | P | P | P | P | S | S | P | S | S | S | S |
| Discomfort using digital devices | **S** | S | S | X | X | S | X | X | S | X | S | X | X | X | X | X | S |
| Reading text on digital devices | X | X | X | **S** | X | X | S | X | S | S | X | X | X | S | X | X | X |
| Difficulty seeing digital screens | X | X | X | X | X | X | X | **S** | X | X | X | S | S | S | X | S | X |
| Limiting use of digital screens | X | **S** | X | X | X | X | X | X | X | X | X | S | X | X | S | X | X |
| Travel | **S** | S | S | NE | S | NE | S | S | S | S | S | S | S | S | S | S | S |
| Driving | **S** | S | S | X | X | X | S | S | S | S | S | S | S | S | S | S | S |
| Use of transport | **S** | S | X | X | S | NE | S | P | NE | X | NE | S | X | S | X | S | X |
| Reading travel schedules | X | X | X | X | **S** | S | S | X | X | X | S | X | NE | S | S | X | X |
| Getting on and off transport | X | **S** | S | X | X | X | X | P | X | X | X | X | X | NE | X | X | X |
| Reading | **S** | S | S | P | S | S | S | P | S | S | S | S | NE | S | NE | S | S |
| Print | **S** | X | S | S | S | X | S | P | S | S | S | X | X | S | X | S | S |
| Handwritten text | P | X | P | X | X | X | X | X | X | **S** | X | X | X | X | X | X | P |
| Text on colored background | X | P | P | X | X | X | X | X | X | **S** | X | X | X | X | X | X | X |
| Activities of daily living | **S** | S | P | P | S | NE | P | S | NE | P | S | S | P | S | P | S | S |
| Shopping | **S** | P | P | X | S | X | P | S | X | P | X | S | P | S | X | X | P |
| Household chores | X | P | **S** | X | P | X | P | S | X | P | X | S | X | P | X | S | X |
| Cooking | X | **S** | S | X | S | X | P | X | X | X | S | X | X | S | X | X | X |
| Personal care/putting on make-up | X | X | **S** | X | X | X | S | P | X | X | S | X | X | S | X | X | X |
| Locating household items | X | X | X | X | **S** | X | X | X | X | X | X | X | X | S | X | X | X |
| Difficulty performing childcare | X | X | **S** | X | X | X | X | X | X | X | X | X | X | X | X | X | X |
| Physical functioning | **S** | P | P | P | P | NE | S | S | P | P | P | S | S | S | P | S | S |
| Using stairs | **S** | S | X | P | S | X | S | S | P | S | P | S | X | S | S | S | P |
| Sports | X | X | X | X | **S** | X | S | X | X | X | X | S | S | X | X | S | S |
| Exercise | X | X | P | X | **S** | NE | X | X | NE | X | NE | S | S | S | X | S | X |
| Balance problems | X | X | X | X | X | X | X | X | X | X | X | **S** | X | X | X | X | X |
| Distal impacts |  |  |  |  |  |  |  |  |  |  |  |  |  |  |  |  |  |
| Work/school | **S** | S | S | NE | S | S | S | P | S | S | S | P | P | S | P | S | S |
| Social functioning | P | **S** | S | S | P | NE | S | S | S | P | NE | S | S | S | P | S | NE |
| Emotional wellbeing | **S** | P | S | NE | P | P | S | S | NE | S | S | S | NE | P | NE | S | P |
| Note. S = spontaneous, P = probed, NE = not experienced and U = unclear. X = not discussed.  *coding: patient number - condition (RP or CHM) – disease severity (MIL= mild, MOD=moderate, SEV=severe as defined in Figure 1) Bold large font represents the first time a concept was mentioned. | | | | | | | | | | | | | | | | | |

**Part 3: Quantitative analysis of symptoms by clinical variables**

**Most commonly reported symptoms (spontaneous and probed) by sum of symptoms**


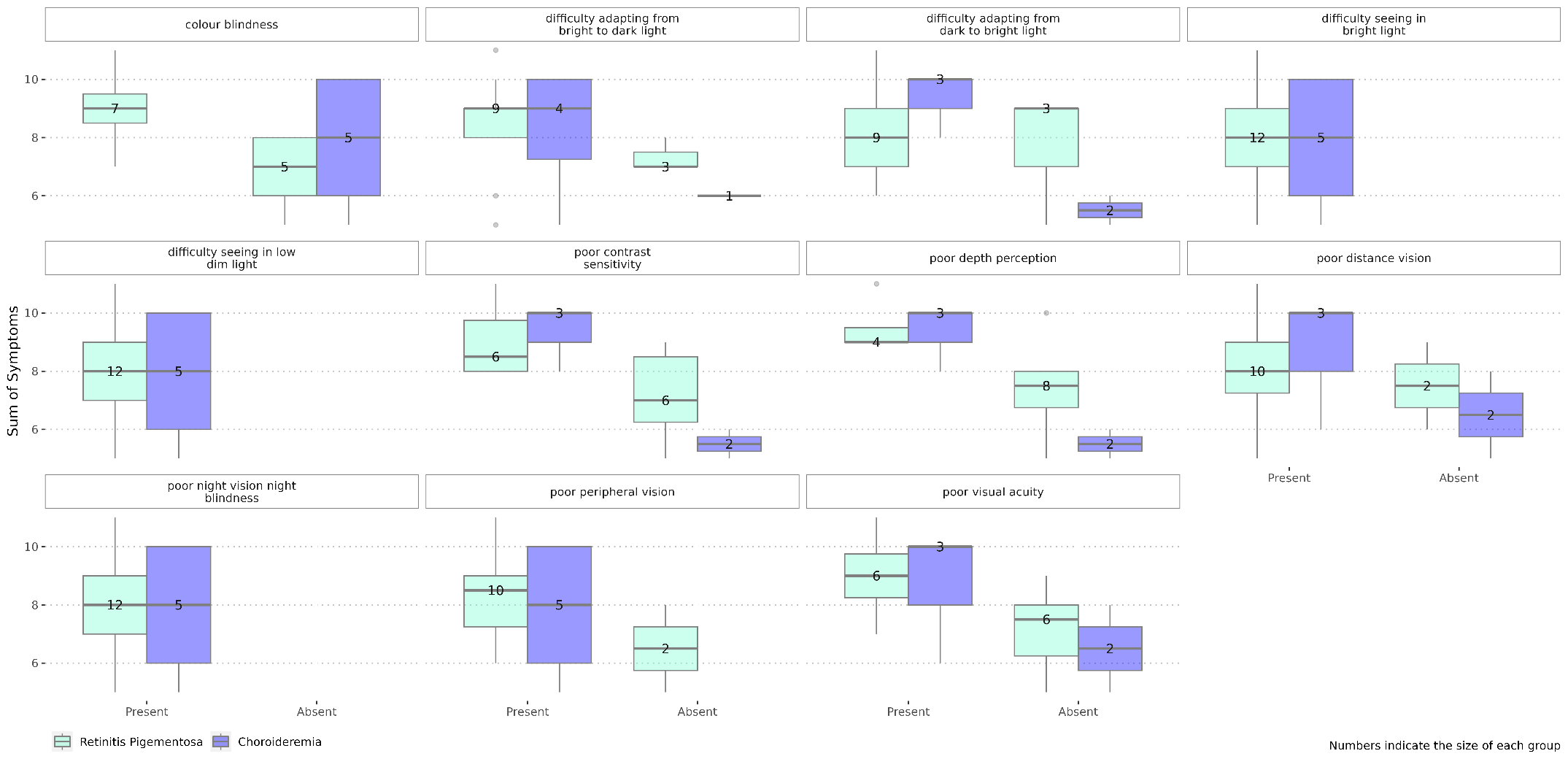


Figure 5: Most commonly reported symptoms (spontaneous and probed) by sum of symptoms

**Symptoms reported (spontaneous or probed) by BCVA (ETDRS letters)**
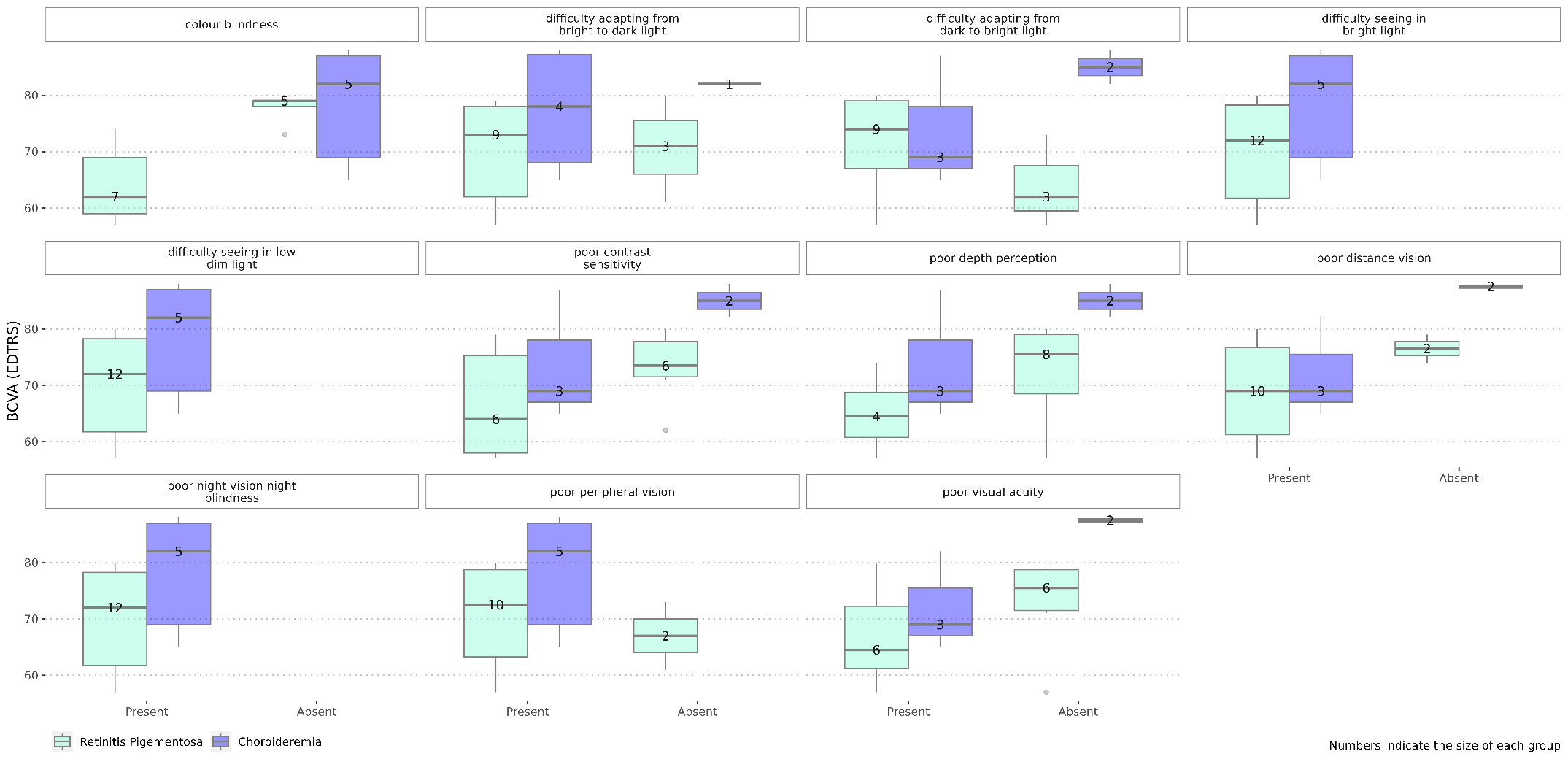


Figure 6: Symptoms reported (spontaneous and probed) by binocular BCVA (ETDRS letters)

Symptoms reported (spontaneous or probed) by Ellipsoid Zone


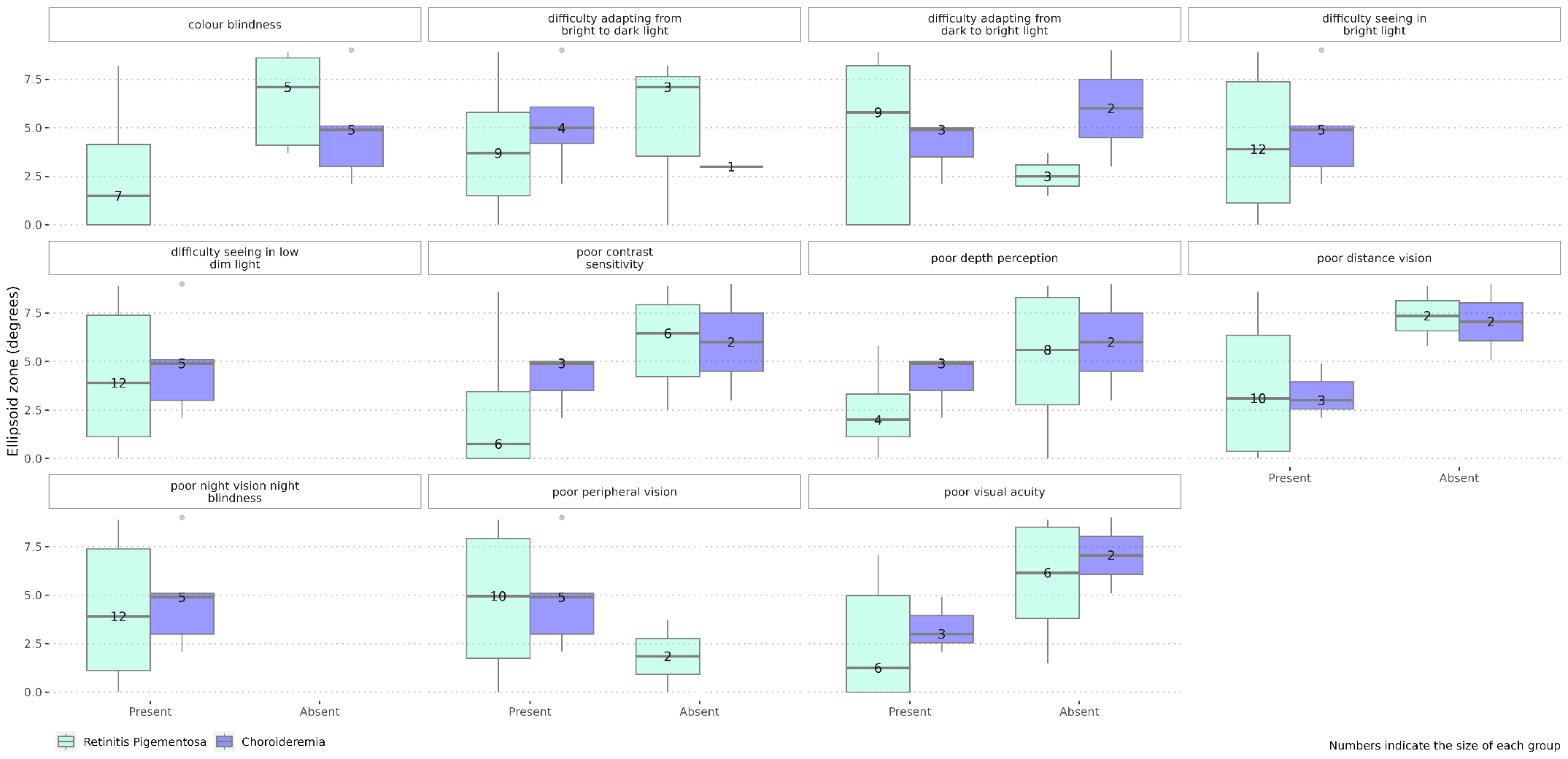


Figure 7: Symptoms reported (spontaneous and probed) by Ellipsoid Zone (in degrees)

Symptoms reported (spontaneous or probed) by Iftikhar cumulative score
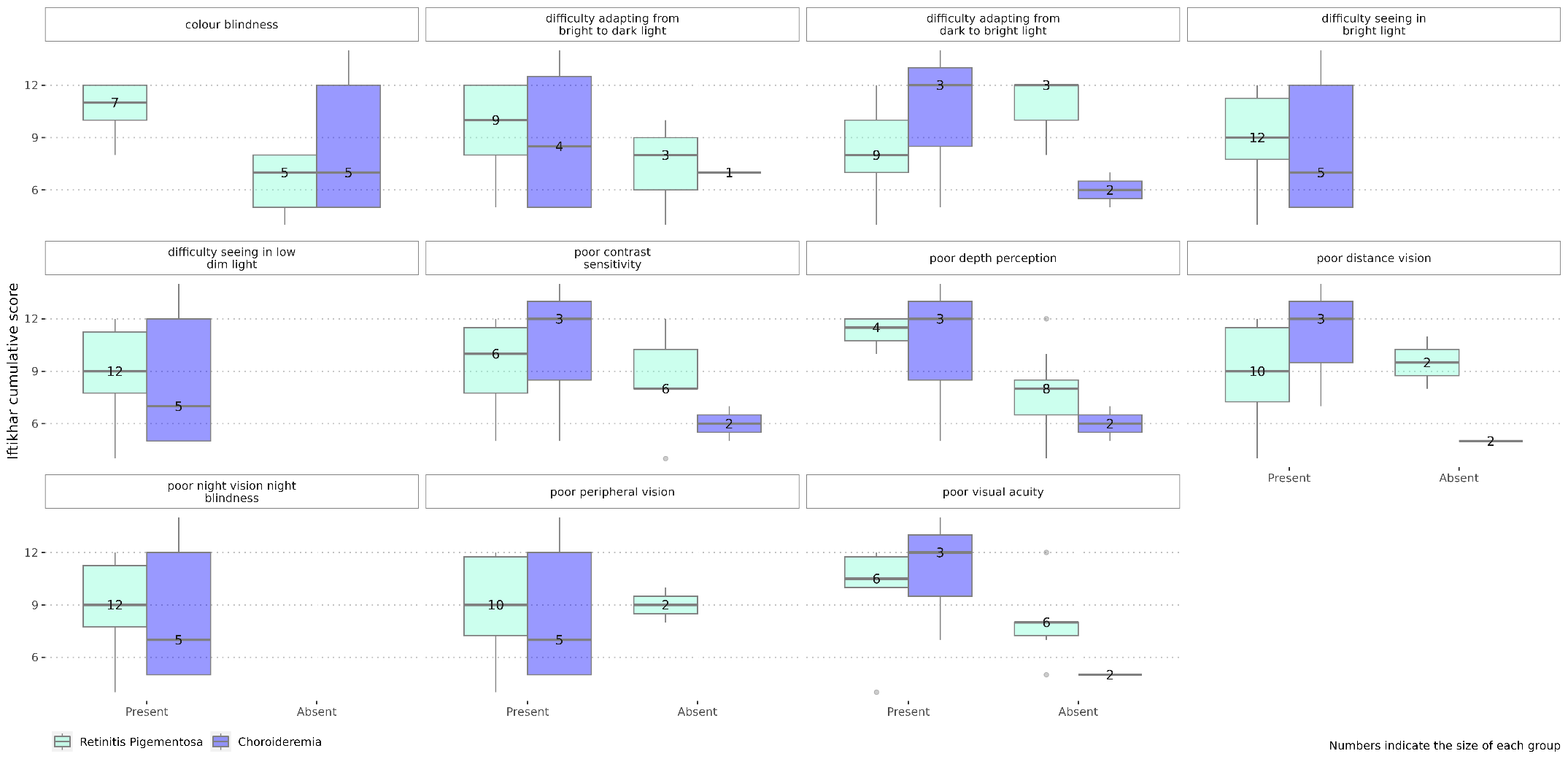


Figure 8: Symptoms reported (spontaneous and probed) by Iftikhar cumulative score

Symptoms reported (spontaneous or probed) by Goldman Visual Field horizontal diameter (degrees)


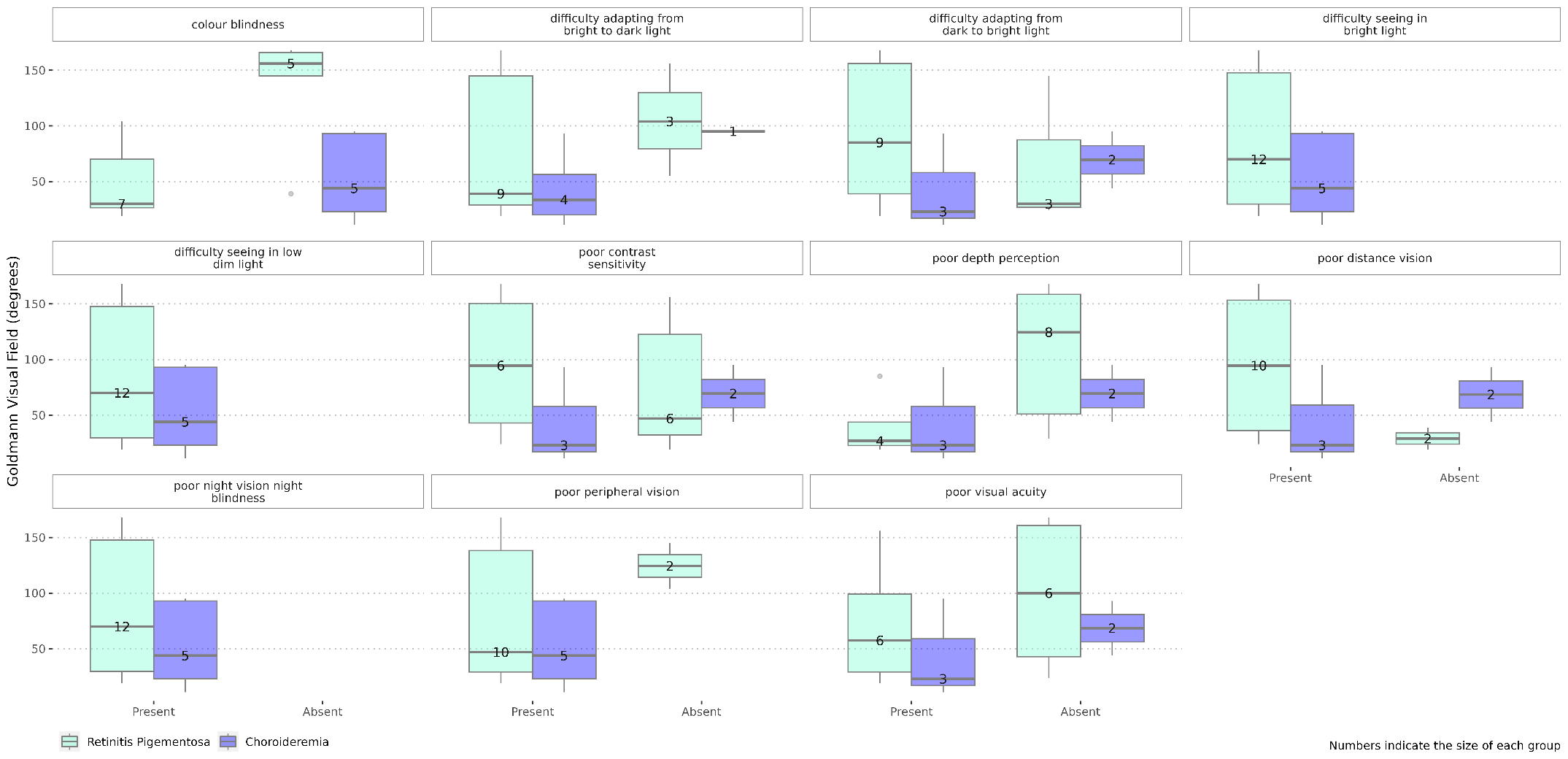


Figure 9: Symptoms reported (spontaneous and probed) by binocular Goldman Visual Field horizontal diameter (degrees)
